# Barriers to Fresh Fruit and Vegetable Access in Adult Patients in the Mayo-La Crosse Family Medicine Residency Clinic

**DOI:** 10.7759/cureus.63114

**Published:** 2024-06-25

**Authors:** Khyati H Patel, Benita N Lin, Jenna Mullins, Alexander S Mortensen, Stephen K Stacey

**Affiliations:** 1 Family Medicine, Mayo Clinic Health System, La Crosse, USA

**Keywords:** lifestyle disease prevention, and practices, attitudes, knowledge, high-fiber diet, barriers for dietary intake, fruit and vegetables

## Abstract

Introduction: Food insecurity remains a pressing issue in the United States, with approximately 12.8% of American households experiencing this challenge in 2023, as reported by the US Department of Agriculture (USDA). In Wisconsin, a state with a notable agricultural heritage, the prevalence of food insecurity averaged 9.9% between 2019 and 2023. A growing body of research underscores the importance of consuming diets rich in fruits and vegetables for maintaining optimal health and mitigating the risk of various chronic diseases, including cardiovascular disease (CVD) and obesity. Fruits and vegetables are reservoirs of essential nutrients such as vitamins, minerals, antioxidants, and dietary fiber, which collectively contribute to overall well-being. Despite the well-documented health benefits of fruits and vegetables, a considerable proportion of the population fails to meet the recommended daily intake of at least five servings. This disparity underscores the importance of exploring factors influencing dietary behaviors and identifying strategies to promote adherence to dietary guidelines.

Methods: Surveys were distributed by staff at the family medicine residency clinic in La Crosse, Wisconsin, during routine visits in April and May 2023. Any patients ≥18 years old presenting to the Family Health Clinic (FHC) were eligible for inclusion. Patients were excluded if they were unable to read/answer survey questions due to intellectual/language/other barriers. Surveys included demographic data such as the participant's age, gender, race, income bracket, and primary mode of transportation. A total of 122 participants were then asked how many servings in a day they ate of 100% juice, fruits, beans, green vegetables, yellow/orange vegetables, and other vegetables, as well as about barriers to more fruit and vegetable consumption and a Likert scale about their attitudes toward fruit and vegetable consumption and interest in discussing it with a healthcare provider. Demographic characteristics were analyzed through graphical representation to elucidate trends and patterns among the surveyed population. We compared different demographics with the barriers to fruits and vegetables using bar graphs.

Results: The primary barrier to fruit and vegetable intake varied by age group: "cost" for 18-30, "other" for 31-50, "cost" and "none" for 51-70, and "none" for over 70. For gender, "none" was most frequent for males while "cost" was for females. By income, "cost" was common for $0-20k and $20-50k, "none" for $50-100k, and "other" for >$100k. A Likert scale assessed interest in discussing healthy foods with healthcare providers. Most responses were "neutral," with "strongly agree" highest in 18-30 and "agree" in 31-50, 51-70, and over 70 age groups.

Conclusions: The purpose of this study was to ascertain barriers to fruit and vegetable access and identify patients' attitudes toward discussing healthy food choices with healthcare providers. There appears to be a correlation between decreasing income and increasing age and the likelihood of identifying cost as a barrier to fruit/vegetable intake. Barriers identified in our clinic included convenience/time constraints and cost. Many people in our survey also identified the lack of quality or good variety of fruits and vegetables at the store (availability) as a significant barrier to eating them.

## Introduction

According to the US Department of Agriculture (USDA), 12.8% of American households experienced food insecurity in 2023. The average rate, from 2019 to 2023, in Wisconsin has been 9.9%. Evidence from multiple large cohort studies suggests that diets high in fruits and vegetables are linked to a decreased incidence of cardiovascular disease (CVD) and obesity [1-4].

Fruits and vegetables contain vital nutrients such as vitamins, minerals, antioxidants, and dietary fiber. In a population-based cohort study from the Netherlands, higher consumption of fruits and vegetables was associated with a lower risk of coronary heart disease (CHD). The risk of CHD was 34% lower for those with a high intake of fruits and vegetables (>475 g/day) compared with those with a low total fruit and vegetable intake (<241 g/day) [3]. A systematic review of fruit and vegetable intake and the incidence of type 2 diabetes showed an association of 14% risk reduction of type 2 diabetes with greater intake of green leafy vegetables [4]. Despite these well-known benefits of fruits and vegetables, only a quarter of patients obtain the recommended intake of at least five fruits and vegetables daily [2].

It may be that access to fresh fruits and vegetables is a significant barrier to those who are seeking to make healthier lifestyle choices. Singleton et al. described a variety of barriers that keep patients from achieving their target for daily fruit and vegetable consumption, including knowledge of cooking, cost, and habits [2]. The concept of barriers to fruit and vegetable consumption extends beyond the overt issue of cost, encompassing a broad spectrum of interconnected challenges that impede individuals' access to and consumption of these essential dietary components. While affordability certainly stands out as a prominent concern, it represents merely the surface-level manifestation of deeper-rooted obstacles such as access to supermarkets, ethnic disparities, and societal norms [5,6]. Several studies have adopted a nuanced approach by treating fruits and vegetables as distinct categories, thereby uncovering specific barriers unique to each entity. This analytical stratification has yielded multifaceted understandings of dietary behaviors, allowing for targeted interventions to address specific barriers associated with fruit and vegetable consumption [7,8].

According to a 2009 systematic review article focusing on food deserts (areas with poor access to healthy food), there were four main barriers to accessing healthy food [5]. The first is access to supermarkets. The study demonstrated that the lowest-income neighborhoods have nearly 30% less supermarkets than the highest-income neighborhoods. Low-income neighborhood residents also face a lack of transportation, a lack of time to go grocery shopping due to work schedules, or a lack of time required to prepare meals. Another barrier is racial and ethnic disparities in food deserts. This study has found that predominantly African American neighborhoods have fewer supermarkets compared with predominantly White neighborhoods, and the distance travelled to and from the nearest supermarket was further for impoverished African American neighborhoods compared with impoverished White neighborhoods. The third barrier found in this systematic review is socioeconomic status [5]. Food prices are higher, and food quality is poorer in areas where poverty is the highest. Finally, they found that urban supermarket prices are higher than suburban ones. There are fewer supermarkets and chain stores that offer cheaper prices compared with smaller grocery stores that tend to stock leading brand items/smaller package sizes, which are more expensive [5].

Another qualitative study was performed in low-income populations in North Carolina to study received barriers to fruit and vegetable consumption. The researchers were able to conduct eight focused groups and identify six major community-level barriers to access to fruits and vegetables, which are the following: cost, transportation, quality, variety, changing food environment, and changing societal norms on food [6].

It is clear that barriers to fruit and vegetable consumption vary by location and socioeconomic makeup. This study aims to understand barriers to fresh fruit and vegetable access in adults living in rural and suburban communities. We developed a survey that was given to patients seeking care in the Mayo-La Crosse Family Health Clinic (FHC) in La Crosse, Wisconsin, a medium-sized referral hospital in a predominantly rural region. We wanted to determine whether there are any correlations among factors such as age, gender, ethnicity, household income, and transportation. We also aimed to identify patients' attitudes around food and willingness to discuss with healthcare providers as a basis for future interventions.

## Materials and methods

Surveys were distributed by staff at the family medicine residency clinic in La Crosse, Wisconsin, during routine visits in April and May 2023 (see Appendices for the survey). Any patients ≥18 years old presenting to the Family Health Clinic were eligible for inclusion. Patients were excluded if they were unable to read/answer survey questions due to intellectual/language/other barriers. The Mayo Clinic Institutional Review Board (IRB) issued approval 22-012364.

Surveys included demographic data such as the participant's age, gender, race, income bracket (annual), and primary mode of transportation, which were created via collaborative brainstorming sessions conducted by our research team. The participants were then asked how many servings in a day they ate of 100% juice, fruits, beans, green vegetables, yellow/orange vegetables, and other vegetables, as well as about barriers to more fruit and vegetable consumption and a Likert scale about their attitudes toward fruit and vegetable consumption and interest in discussing it with a healthcare provider. For the study, the Behavioral Risk Factor Surveillance System (BRFSS) was employed to ascertain the respondents' daily consumption of fruits and vegetables. Specifically, the survey inquired about the number of servings consumed per day [9]. Guidelines established by the US Department of Agriculture (USDA) pertaining to fruit and vegetable intake, stratified by age and gender, were utilized as reference points for assessing adherence to recommended dietary standards [10]. A Likert scale was developed to gauge the respondents' attitudes, perceptions, or opinions toward fruit and vegetable consumption and interest in discussing it with a healthcare provider. The respondents were asked to indicate the extent of their agreement or disagreement on a predetermined scale. This scale typically ranges from "strongly disagree" to "strongly agree." The quantitative data were analyzed to discern patterns or trends within the respondents' perspectives.

One hundred twenty-two surveys were administered, and data were compiled into a Microsoft Excel sheet (Microsoft Corp., Redmond, WA) by demographics and compared with each other. Demographic characteristics were analyzed through graphical representation to elucidate trends and patterns among the surveyed population. We compared different demographics with the barriers to fruits and vegetables using bar graphs. It is pertinent to note that the data analysis phase refrained from extensive statistical manipulation due to the complex nature of the variables under investigation and the inadequacy of statistical power. The determination of an appropriate sample size was executed using an online sample size calculator (www.calculator.net/sample-size-calculator), which computes the minimum number of necessary samples to meet the statistical constraints, predicated on the adult patient population within the Family Health Center (FHC). This approach facilitated the establishment of a sample size deemed sufficient for the study's objectives.

## Results

Survey responses were collected from 122 patients. Of the respondents, 39 were between the ages of 18 and 30, 32 were between the ages of 31 and 50, 33 were between the ages of 51 and 70, and 18 were over the age of 70. Regarding gender, 29 were male, 91 were female, and two were nonbinary/omitted their gender. As for household income, 29 individuals had a household income between $0 and $20k, 21 individuals had between $20k and $50k, 38 individuals had between $50k and $100k, 25 had over $100k, and nine had unsure/omitted their income (Table 1).

**Table 1:** Demographic data

Demographics	Number of participants
Age, 18-30 years	39
Age, 31-50 years	32
Age, 51-70 years	33
Age, >70 years	18
Females	91
Males	29
Nonbinary/omitted gender	2
Household income between $0 and $20k	29
Household income between $20k and $50k	21
Household income between $50k and $100k	38
Household income over $100k	25
Unsure/omitted income	9

**Figure 1:**
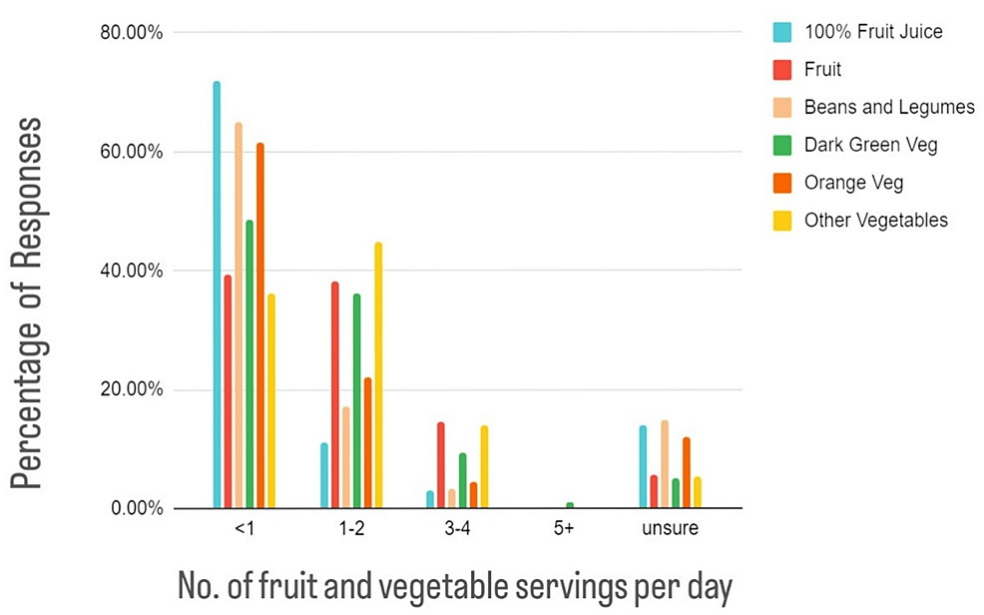
Self-reported servings of fruits and vegetables per day These answers were given in response to the question, "Over the past month, how many times per day did you eat?" with answers including 100% fruit juice, fruit, beans and legumes, dark green vegetables, orange vegetables, and other vegetables. The most common response in all categories of fruits and vegetables was <1 serving per day with the most common type of vegetable consumption being "other vegetables" including tomatoes, cabbage, and potatoes

The most common response in all categories of fruits and vegetables was <1 serving per day with the most common type of vegetable consumption being "other vegetables" including tomatoes, cabbage, and potatoes (Figure 1).

**Figure 2:**
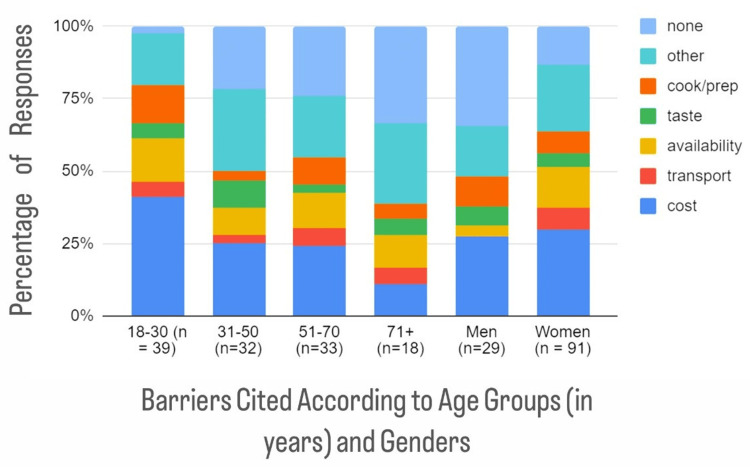
Self-reported barriers to increasing fresh fruit/vegetable intake according to age groups (in years) and gender These answers were given in response to the question, "What do you think is a barrier to increasing fresh fruit/vegetable intake in your diet?"

The most frequently cited barrier to the intake of fruits and vegetables was "cost" for those aged 18-30, "other" for those aged 31-50, "cost" and "none" for those aged 51-70, and "none" for those older than 70. Regarding barriers by gender, "none" was the most frequent barrier for males, while "cost" was the most frequent barrier for females (Figure 2).

**Figure 3:**
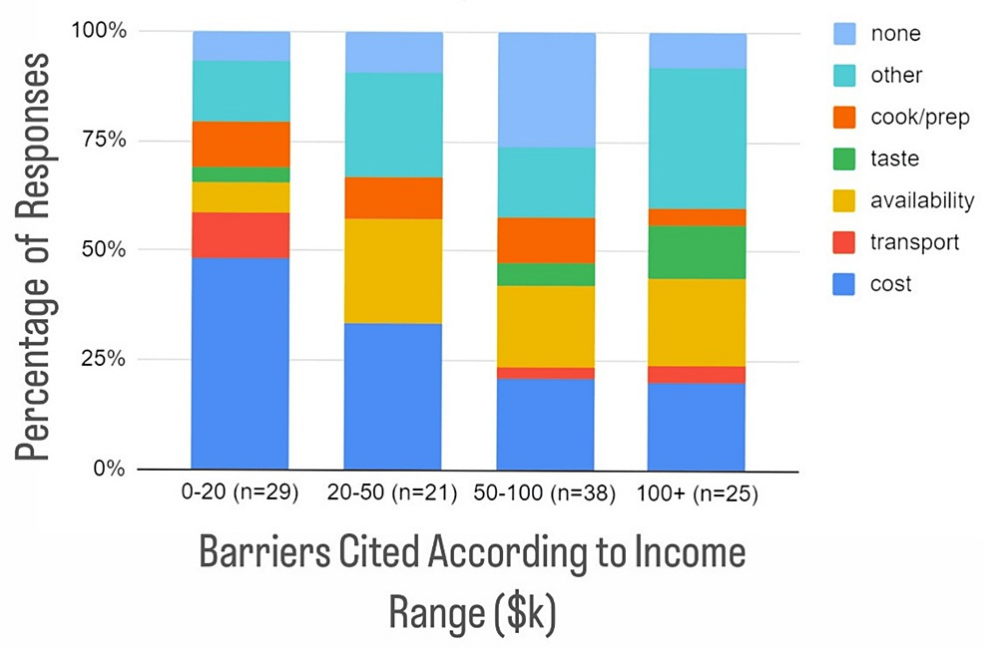
Self-reported barriers to fruit and vegetable intake by income range ($k) Cost became a less identified factor with increasing income (48% of individuals with household incomes of $0-20k identified "cost," while only 20% of individuals with household incomes of >$100k selected "cost"). Few households making <$20k/year identified variety/quality as a barrier

In reference to barriers by income, the most frequent barrier was "cost" for household incomes between $0 and $20k, "cost" for household incomes between $20k and $50k, "none" for household incomes between $50k and $100k, and "other" for household incomes >$100k (Figure 3).

**Figure 4:**
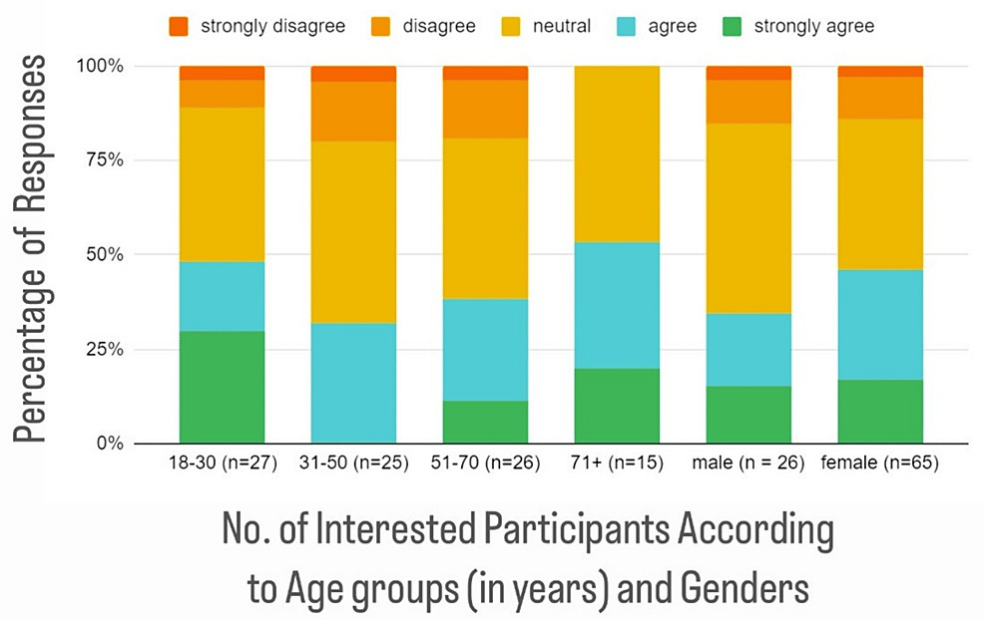
Interest in discussing nutrition with providers by age groups (in years) and gender Twenty-six out of 29 males and 65 out of 91 females responded to this question. Forty-three percent of the respondents either agreed or strongly agreed that they would be interested in speaking with a provider about their diet, with another 41% neutral. There was not a large difference between age groups and genders

A Likert scale was obtained to assess patient interest in discussing healthy foods with healthcare providers. Most responses in all age groups were "neutral," followed by "strongly agree" in those 18-30 years old, "agree" in those 31-50 years old, "agree" in those 51-70 years old, and "agree" in those older than 70 years old (Figure 4).

## Discussion

The purpose of this study was to ascertain barriers to fruit/vegetable access and identify patients' attitudes toward discussing healthy food choices with healthcare providers. The most frequently cited barrier was cost, which became less commonly selected with increasing age, while "none" became more common. The next most selected factor was "other." Out of 28 individuals who chose "other," 26 provided responses. The most common factors were "time to prepare" and "food intolerances" (five each), with two mentioning spoilage/waste. "Cost" was less chosen with higher income. Insufficient surveys hindered linear regression analysis yet implied a potential correlation between income and perceived cost barriers to fruit and vegetable intake.

A 2011 study, conducted via computer-assisted telephone interviews, provided an alternative perspective. It uncovered significant differences in consumption patterns, barriers, cognitive awareness, and readiness for change between fruits and vegetables [7]. While cost was the main obstacle for fruit consumption, food preferences were the primary barrier for vegetables. Supporting this, an online survey in New South Wales revealed distinct barriers: fruit spoilage and vegetable quantity perception. These findings underscore the need for varied strategies to improve the consumption of both food groups [8].

In addition to the economic factor of cost, various barriers such as taste preferences, inconvenience, forgetfulness, time constraints, dissatisfaction of appetite, a lack of motivation, and insufficient knowledge of intake recommendations and preparation methods emerge as primary barriers to the increased consumption of fruits and vegetables [11-13]. Notably, an observed trend among college students emphasizes specific challenges, with a significant percentage citing barriers such as the widespread availability of fast food, limited time, and lethargy as key hindrances to fruit and vegetable consumption within this demographic [12,13]. Addressing these multifaceted barriers necessitates tailored strategies, acknowledging the unique challenges faced by distinct populations.

To overcome barriers, a range of strategies can be implemented at various levels, including individual to national scales. These encompass multicomponent interventions such as behavior change communication (BCC), nutrition education (NE), gardening programs, farm-to-institution programs (FIPs), food assistance initiatives such as baskets and cash transfers, and policies linking nutrition and agriculture [14]. A study suggests prioritizing fruit and vegetable intake as a habitual practice in health promotion programs for low-income older adults, aiming to translate intentions into sustained dietary habits [15]. These insights inform future interventions and public health initiatives to promote healthier dietary practices.

Of course, this study suffers from the usual limitations of survey research, including recall bias, social acceptability bias, and in-group bias. Also, there may be important barriers that were not reflected in the question set we used, prompting "other" to be a commonly selected option. Also, we cannot assert a definitive identification of the actual barriers. Rather, these results encapsulate patients' expressed difficulties in the context of a primary care visit. Other limitations of this study include being underpowered. Based on the initial power calculation with our clinic size (approximately 7000 adult patients, aged 18 and over), the sample size needed to have a confidence interval of 95% with a margin of error of 5%, which was 368, and 122 surveys were collected. Based on the number of surveys collected, there was a confidence interval of 95% with a margin error of 9%. Despite these limitations, our findings are consistent with prior reports of barriers to fruit and vegetable intake.

The data were collected using a convenience sample, which means that many surveyed individuals did not have any barriers to report. While this is more representative of the clinic population in general, it may not reflect populations with markedly different levels of food insecurity. In addition, several surveys were filled out incompletely. While this was expected (patients were notified that they may skip any questions that they did not feel comfortable answering), it also may create unaccounted biases in the data. Some patients wrote comments on the sides of the survey that the questions were unclear.

Patients in our clinic are not meeting the recommended fruit and vegetable intake, even those who do not perceive barriers or consider their food choices healthy. While 93% acknowledge the link between increased intake and reduced heart disease risk and 87% recognize the association with lower diabetes risk, consumption does not align with these beliefs. Future studies might explore beliefs around cooking and eating habits through focus groups or interviews to develop interventions aligning with patients' preferences and increasing intake.

## Conclusions

This survey of patients in a primary care setting revealed that the primary barriers were convenience/time constraints and cost. Lower income and older age were correlated with identifying cost as a barrier. The results of this survey can help guide decisions about how to improve access to healthy food choices.

## Figures and Tables

**Figure 5:**
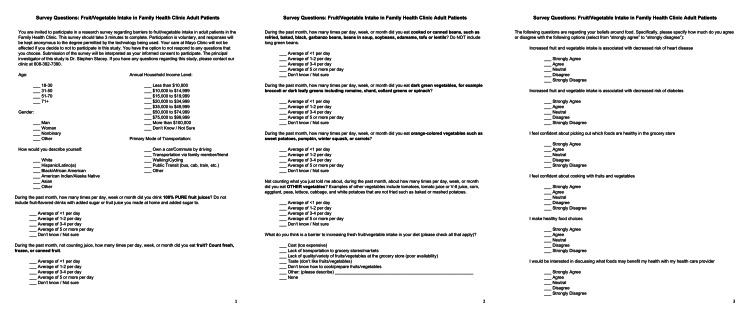
Survey

## Appendices

Figure 5 shows the survey questions.
